# Hysteria in Children

**Published:** 1907-04

**Authors:** 


### HYSTERIA IN CHILDREN.

Hecht gives the treatment in hysteria in children as follows: Just as hysterical symptoms are ushered in with slight provocation, so they may be made to disappear with slight but appropriate treatment. As soon as the nature of the ailment stands revealed to the physician the parents must be made to understand the crux of the situation and be entirely governed by it. They must be told that a dispassionate interest, or, better, no interest at all, is the most unwholesome thing for the child. Moral suasion is desirable, especially in cases with purely psychic manifestations, and, together with a firm yet gentle order of discipline, initiated early, will result in great and lasting good. When one is denied the intelligent and obedient co-operation of parents, and this is only too often the case, isolation becomes an imperative measure. Isolation to be complete and effective means no visitors, no letters, no messages; in short, no reminders of the past. With no intent to deprecate the talent and skill of the trusted family physician, the author thinks that he is not the best nor, as a rule, the last advisor in hysterical cases. This disease is not a grateful one for the family physician to treat. Strange surroundings, strange people, and the strangest physician will exert the greatest good and effect the quickest cure. The principle involved in the method of surprise (Ueberrumpelungs Methode of Bruns) is at once simple and logical. To emancipate the child from a deep rooted obsession and do it quickly and completely (in one trial, if possible) is the purpose of the method. Give such children a chance to reflect and deliberate, and it means almost certain defeat for the cure. In conjunction with isolation and these clever mental devices, all methods of avail in adult functional diseases are of use here, such as diet, massage, electricity, the lukewarm bath and cold spinal douche, or the cold drip sheet. Hydrotherapy and electrotherapy are in the main extremely unpleasant and in part painful, hence successful. Hypnotism has met with growing disfavor, and is by many regarded as dangerous to children, if for no other reason that its being a form of induced hysteria. After a cure in isolation has been effected, children should not be returned to their old surroundings too soon. They should be given plenty of time in which to forget all the circumstances attending their former disabilities.—Jour. A. M. A., February 23d, 1907.

